# Cost-effectiveness of WEB Embolization, Coiling and Stent-assisted Coiling for the Treatment of Unruptured Intracranial Aneurysms

**DOI:** 10.1007/s00062-023-01311-0

**Published:** 2023-06-27

**Authors:** Lukas Goertz, Julia Simões Corrêa Galendi, Christoph Kabbasch, Marc Schlamann, Lenhard Pennig, Matthias F. Froelich, Marco Timmer, Thomas Liebig, Stephanie Stock, Dirk Mueller, Nils Große Hokamp

**Affiliations:** 1 Faculty of Medicine and University Hospital, Department of Radiology and Neuroradiology, University of Cologne, Kerpener Straße 62, 50937 Cologne, Germany; 2 Institute of Health Economics and Clinical Epidemiology, Faculty of Medicine and University Hospital of Cologne, University of Cologne, Kerpener Straße 62, 50937 Cologne, Germany; 3 Department of Radiology and Nuclear Medicine, University Medical Centre Mannheim, Mannheim, Germany; 4 Faculty of Medicine and University Hospital, Center for Neurosurgery, University of Cologne, Kerpener Straße 62, 50937 Cologne, Germany; 5 Faculty of Medicine and University Hospital, Department of Neuroradiology, University of Munich (LMU), Marchioninistraße 15, 81377 Munich, Germany

**Keywords:** Cost-effectiveness, Health care, Neuroembolization, Woven Endobridge

## Abstract

**Purpose:**

Information about the cost-effectiveness of a certain treatment is relevant for decision-making and healthcare providers. This study compares the cost-effectiveness of the novel Woven Endobridge (WEB) for intracranial aneurysm treatment with conventional coiling and stent-assisted coiling (SAC) from the perspective of the German Statutory Health Insurance.

**Methods:**

A patient-level simulation was constructed to simulate 55-year-old patients with an unruptured middle cerebral artery aneurysm (size: 3–11 mm) considering WEB treatment, coiling or SAC in terms of morbidity, angiographic outcome, retreatment, procedural and rehabilitation costs and rupture rates. Incremental cost-effectiveness ratios (ICERs) were calculated as costs per quality-adjusted life years (QALYs) and costs per year with neurologic morbidity avoided. Uncertainty was explored with deterministic and probabilistic sensitivity analyses. The majority of data were obtained from prospective multi-center studies and meta-analyses of non-randomized studies.

**Results:**

In the base case, lifetime QALYs were 13.24 for the WEB, 12.92 for SAC and 12.68 for coiling. Lifetime costs were 20,440 € for the WEB, 23,167 € for SAC, and 8200 € for coiling. Compared to coiling, the ICER for the WEB was 21,826 €/QALY, while SAC was absolutely dominated by WEB. Probabilistic sensitivity analysis revealed that at a willingness-to-pay of ≥ 30,000 €/QALY, WEB was the preferred treatment. Deterministic sampling showed that the discount rate, material costs and retreatment rates had the largest impact on the ICERs.

**Conclusion:**

The novel WEB showed at least comparable cost-effectiveness to SAC for treatment of broad-based unruptured aneurysms. Considering all three modalities, coiling had the least costs; however this modality is often not appropriate for the treatment of wide-necked aneurysms.

**Supplementary Information:**

The online version of this article (10.1007/s00062-023-01311-0) contains Supplementary Information, which is available to authorized users.

## Introduction

Because of the widespread use of imaging modalities, unruptured intracranial aneurysms (UIAs) are detected with increasing frequency. In Germany, the number of patients who were admitted to hospital for an UIA increased by a factor of 2.3 from 2005 to 2017, with a large proportion of patients being older than 69 years [9].

The rupture of an intracranial aneurysm leads to subarachnoid hemorrhage (SAH), which is associated with high morbidity and mortality [17]. The overall incidence of aneurysmal SAH is approximately 9/100,000 people per year [17]. The appropriate management of UIAs depends on the individual risk factors for aneurysm rupture and the anticipated individual risk of treatment-related complications. Over the past two decades, endovascular treatment has evolved as the first-line therapy for UIAs and various devices and endovascular techniques have been developed since then.

Conventional coiling represents the long-term standard technique for endovascular aneurysm treatment. Stent-assisted coiling (SAC) and flow-diverters allow the treatment of morphologically complex aneurysms and can provide higher aneurysm occlusion rates than conventional coiling. However, these techniques are associated with an increased risk for ischemic stroke [19]. Since the introduction of the Woven Endobridge (WEB) in 2011, intrasaccular flow-disruption has evolved as a proven concept for endovascular aneurysm therapy, in particular for the treatment of wide-necked bifurcation aneurysms. A further benefit of WEB over stent-assisted procedures is that it does not require long-term anti-platelet medication.

Besides clinical and angiographic outcomes, the economic impact of the respective endovascular technique may be relevant for health care decision makers and health care providers. Previous cost analyses revealed that the net material costs (e.g., stent or WEB) highly impact the overall hospital costs. However, little is known about the long-term economic impact of the WEB device for treatment of UIAs, in particular considering the overall treatment costs and long-term health benefits. The objective of this modeling analysis was to compare the cost-effectiveness of WEB treatment with SAC for the treatment of wide-necked unruptured aneurysms from the perspective of the German Statutory Health Insurance (SHI). The cost-effectiveness of conventional coiling was also calculated for reference, although wide-necked aneurysms are usually not treated with this method.

## Methods

A patient-level simulation with a life-long time horizon and a 6-month cycle length was developed to reflect and compare the clinical and economic long-term consequences of coiling, SAC and WEB. The cycle length was chosen to reflect the potential angiographic and clinical events on the first year of follow up. All model calculations were performed with TreeAge Pro 2019 (TreeAge Software, LLC, Williamstown, MA, USA). The model patients were assumed to have a single UIA located at the middle cerebral artery (MCA) with saccular shape and an aneurysm diameter between 3 and 11 mm. In line with recent epidemiological evidence from Germany [9], patients of the model cohort were assumed to be 55 years old (standard deviation: 10.2 years), at a proportion of 67% female and 33% male. WEB patients were assumed to receive acetylsalicylic acid (ASA) monotherapy 100 mg/day for 6 weeks [16]. In SAC, we assumed a daily antiplatelet regimen with ASA 100 mg life-long and clopidogrel 75 mg for 4 months after the procedure. The analysis was performed from the perspective of the SHI, which covers 87% of the German population [4].

### Model Overview

**Fig. 1:**
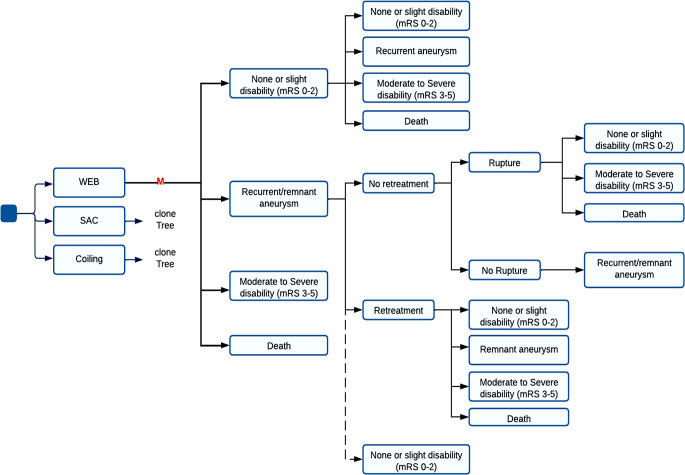
Model overview. *M* indicates start of microssimulation; *dashed line* represents the possibility of a progressive occlusion (transition was possible only in the first 6‑month cycle) (*WEB* Woven Endobridge, *SAC* stent-assisted coiling, *mRS* modified Rankin Scale)

The simulation model incorporated four clinical events which may occur to patients with UIAs after endovascular treatment: i. complete aneurysm occlusion and good clinical outcome (modified Rankin Scale (mRS) 0–2); ii. incomplete occlusion; iii. procedure-related complications leading to a moderate-to-severe disability (mRS 3–5); or iv. procedure-related death. Progressive aneurysm occlusion was considered to occur in the first 6 months. Recurrence (i.e., aneurysm regrowth or recanalization after a successful aneurysm occlusion) was assumed to occur in the first five years after the first treatment. Coiling was considered as retreatment option for remnant or recurrent aneurysms, independent of the initial treatment. After SAH, the outcome scenarios were: i. good outcome (mRS 0–2), ii. moderate-to-severe disability (mRS 3–5), or iii. death. Figure 1 shows an overview of the model.

### Input Parameters

To identify appropriate input parameters for the model, several systematic literature searches were performed in Medline via PubMed. Studies on UIAs with the highest methodological quality were selected, and for ensuring representativeness, data reflecting the German population was preferred whenever possible. Table 1 lists all input parameters and the respective distributions.

**Table 1:** Input Parameters of the base-case patient-level simulation

Parameter	Value	Distribution	Source
***Event probabilities***	6‑m probability (SD)	–	–
**WEB**		–	–
Procedure morbidity (leading up to mRS score > 2)	0.013 (0.009)	β	[31]
Procedure-related mortality	0.007 (0.006)	β	[31]
Adequate occlusion after procedure	0.529 (0.040)	β	[31]
Progressive occlusion	0.618 (0.04)	β	[31]
Recurrence	0.063 (0.02)^a^	β	[31]
Retreatment	0.034 (0.01)	β	[31]
**SAC**		β	–
Procedure morbidity (leading up to mRS score > 2)	0.041 (0.007)	β	[30]
Procedure-related mortality	0.014 (0.002)	β	[30]
Adequate occlusion after procedure	0.576 (0.010)	β	[30]
Progressive occlusion	0.299 (0.02)	β	[30]
Recurrence	0.062 (0.01)^a^	β	[30]
Retreatment	0.062 (0.01)	β	[12, 26]
**Coiling**		β	–
Procedure morbidity (leading up to mRS score > 2)	0.035 (0.004)	β	[30]
Procedure-related mortality	0.001 (0.0001)	β	[30]
Adequate occlusion after procedure	0.487 (0.012)	β	[30]
Progressive occlusion in 6 months	0.175 (0.016)	β	[30]
Recurrence	0.130 (0.02)^a^	β	[30]
Retreatment	0.130 (0.02)	β	[26]
**Retreatment (coiling for all groups)**		β	–
Procedure morbidity (leading up to mRS score > 2)	0.027 (0.015)	β	[38]
Procedure-related mortality	0.001 (0.0001)	β	[30]
Adequate occlusion after procedure	0.487 (0.012)	β	[30]
Probability of a second retreatment	0.025 (0.015)	β	[38]
**Probability of rupture of remnants or recurrent aneurysm**	0.095 (0.066)	β	[13]
**Probability of poor outcome after rupture (mRS 3–5)**	0.181 (0.009)	β	[13]
**Probability of a good outcome after rupture (mRS 0–2)**	0.718 (0.011)	β	[13]
**SAH mortality**	0.09 (0.007)	β	[13]
**Probability of bleeding due to long-term low dose aspirin**	0.00048 (0.003)	–	[14]
***Costs***	Value (SD)	–	–
WEB material costs	€ 11,470 (2294)	γ	–
SAC material costs	€ 11,897 (3857)	γ	–
Coiling material costs	€ 680 (367)	γ	–
DRG (WEB and SAC)	€ 4960	γ	[25]
DRG (Coiling)	€ 2513	γ	[25]
Acute in-hospital SAH treatment	€ 19,823 (3964)	γ	[35]
Consequential in-hospital SAH treatment	€ 4055 (811)	γ	[35]
Rehabilitation	€ 12,499 (2500)	γ	[35]
Home care costs	€ 8429 (1686)	γ	[35]
Digital subtraction angiography (inpatient procedure)^b^	€ 1059 (211)	γ	[25]
Magnetic resonance angiography (outpatient procedure)^b^	€ 78 (15)	γ	[25]
Acetylsalicylic acid monotherapy	€ 4 per month	γ	[1]
Clopidogrel monotherapy	€ 87 per month	γ	[1]
***Utilities***	Value (SD)	–	–
**Age-adjusted utility values for healthy population**	18–24 y: 0.96 (0.06), 25–34 y:0.96 (0.05), 35–44 y: 0.96 (0.07), 45–54 y: 0.95 (0.07), 55–65 y: 0.95 (0.09), 65–74 y: 0.95 (0.10), > 75 y: 0.89 (0.16)	β	[18]
**Utility values for harboring an untreated aneurysm**	0.81 (0.19)	β	[24]
**Utility decrements for bleeding events associated with antiplatelet therapy**	0.029 (0.0048)	β	[8]
**Utility values after SAH according to outcome**		β	[33]
*Good outcome*			
mRS 0	0.93 (0.15)		
mRS 1	0.86 (0.15)		
mRS 2	0.68 (0.20)		
*Bad outcome*			
mRS 3	0.56 (0.23)		
mRS 4	0.31 (0.25)		
mRS 5	0.06 (0.21)		
**Proportion of patients in each mRS 6 months after SAH**^b^		β	[28]
*Good outcome*			
mRS 0	0.25 (0.05)		
mRS 1	0.39 (0.07)		
mRS 2	0.35 (0.07)		
*Bad outcome*			
mRS 3	0.61 (0.12)		
mRS 4	0.15 (0.03)		
mRS 5	0.23 (0.04)		
**Proportion of patients in each mRS after 1 year of SAH**^b^		β	[28]
*Good outcome*			
mRS 0	0.30 (0.06)		
mRS 1	0.37 (0.07)		
mRS 2	0.31 (0.06)		
*Bad outcome*			
mRS 3	0.64 (0.12)		
mRS 4	0.19 (0.03)		
mRS 5	0.16 (0.03)		

*WEB* Woven Endobridge, *SAC* stent-assisted coiling, *mRS* modified Rankin scale, *m* months, *SAH* subarachnoid hemorrhage, *DRG* diagnosis-related group

^a^Recurrence rate was assumed to decrease linearly until reaching 0 on year 6

^b^40% standard deviation was assumed.

#### Event Probabilities

Probabilities of successful aneurysm occlusion, progressive aneurysm occlusion, procedure-related mortality, procedure-related morbidity, aneurysm recurrence and retreatment after endovascular treatment were mainly taken from two studies [30, 31]: For the WEB, Pierot et al. reported the 1‑year follow up of 168 patients based on three single-arm, prospective, consecutive multicenter studies [31]. For coiling (29,388 patients) and SAC (2696 patients), Phan et al. pooled the results of 14 predominantly retrospective studies in a series of meta-analyses [30]. Flow-diverter studies were not included.

A long-term follow-up study of patients after endovascular aneurysm treatment demonstrated that 19% of retreatments are performed within 12 months and 53% during years 1–5 [12]. Hence, the recurrence rates were assumed to decrease linearly for the first five years across all treatment groups (Fig. S1). The lifelong probability of retreatment per strategy is summarized in supplementary Table S1.

The probability of rupture among recurrent aneurysms (3.2%) was taken from a single-center cohort that included 426 unruptured aneurysms with a mean follow-up of 74 months treated from 2009 to 2017 [13]. Aneurysm remnants were assumed to carry a residual risk for aneurysm rupture, while neck remnants were not prone to rupture [29]. The morbidity risk of retreatment was taken from a single-center, retrospective series with 111 cases that were initially treated with coiling or SAC [38]. These data were also used for WEB treatment, as there are only few case series for retreatment after WEB implantation available that do not allow systematic derivation of retreatment risks and retreatment-related morbidity [22]. The risk of bleeding due to long-term antiplatelet therapy were taken from a meta-analysis of observational studies including patients in long-term use of low-dose aspirin, independent of the clinical indication [14].

Patients with unruptured aneurysms were assumed to have the same probability of death as the general population (all-cause mortality, adjusted for age and gender). For patients with a disability (mRS ≥ 2), an excess long-term mortality rate of 17% at 20 years and 32% at 30 years was assumed [11, 20]. For patients with ruptured aneurysms and a good outcome one year after a SAH, a 20% excess mortality compared to the general population was assumed (starting 8 years after the event) [20].

#### Utilities

Utility values for the German healthy population were elicited from a representative sample of 4498 patients with one comorbidity. For the measurement, the EuroQoL-Dimension (EQ-5D) questionnaires and the Visual Analogue Scale were used [18].

Utilities for patients with a moderate-severe disability were taken from a meta-analysis including nine studies (9607 patients) that derived the utility-weighted modified Rankin Scale from the EQ-5D [33]. The utility values were anchored to the proportion of patients in each mRS score, considering that the mRS scores improve in the first year after SAH due to rehabilitation [2, 28]. Lastly, disutility was applied in case of a bleeding resulting from long-term antiplatelet therapy [8]. To combine utility values the multiplicative method was used.

#### Costs

In Germany, hospitals are reimbursed for inpatient procedures mainly via diagnosis-related group (DRG)-based payments [37]. Because the time-lag until an innovative technology is integrated in the DRG scheme may take up to three years, additional funding (i.e., innovation payments) can be negotiated between hospitals and insurers. To reflect the additional costs, the costs of the procedures were calculated by adding the lump-sums according to the respective DRGs and the additional material costs. To calculate the material resource use for SAC and coiling procedures we conducted a micro-costing study with data from the authors’ institution. For this purpose, we retrospectively collected the data of 139 patients with 144 aneurysms sized 3–11 mm and treated with either coiling or SAC and retrieved the individual number of implanted stents and coils (Supplementary Table S2, S3). For WEB, we assumed that one WEB device was used per patient, as suggested in the manufacturer’s instructions for use. The mean costs were calculated weighting the material resource consumption by the unitary costs.

Follow-up costs after treatment consisted of imaging costs (i.e., digital subtraction angiography for WEB and SAC, MR angiography for coiling) 6 and 12 months after treatment and once every two years for the following years [39]. Follow up costs were considered for patients with none or slight disability (mRS 0–2) and a proportion of patients with moderate-severe disability (mRS 3 only). Follow-up costs also included acetylsalicylic acid (ASA) and clopidogrel 75 mg, if applicable.

The costs of treatment for a ruptured aneurysm case were derived from a cost-of-illness study conducted with 101 patients treated for aneurysmal SAH in Germany in 2017 [34, 35]. It was assumed that 60% of cases were treated with coiling and 40% with clipping [42, 43]. Home care-related cost continued to incur annually for a proportion (32%) of patients in the moderate-severe disability state [35]. All costs were adjusted for the year 2021.

### Model Outcomes

The model outputs were measured as incremental cost-effectiveness ratios (ICER), expressed as Euros (€) per years with neurological morbidity avoided (neurological morbidity defined as mRS > 2) for the cost-effectiveness analysis, and € per quality adjusted life-year gained (QALY) for the cost-utility analysis. Costs and outcomes were discounted at a rate of 3% and a half cycle correction was not applied [21, 36].

### Model Validation and Sensitivity Analyses

To validate the model, we consulted experts on the adequacy of input data and the conceptual appropriateness of the model. Technical accuracy was checked regarding data entry and programming errors. For cross model validation, we compared our assumptions to those in similar models. The validation efforts are reported in detail in the supplementary material, following the ‘Assessment of the Validation Status of Health Economic decision models’ checklist [41]. To explore model uncertainty resulting from input parameters, one-way deterministic sensitivity analyses were conducted for selected parameters.

A probabilistic sensitivity analysis (PSA) was conducted to quantify the level of confidence in the output of the analysis. In PSA, input parameters were drawn by random sampling from each distribution, and 10,000 iterations of the model were ‘run’ to generate a distribution of outputs. For the PSA, gamma distributions were used for modelling cost parameters, while probabilities and utilities were assumed to be beta distributed (Table S4; [3]). A distribution was applied to DRG, because although these are fixed values, there is a minimum variation in reimbursement lump-sum depending on duration of hospital stay (1–2 days). In our model, coiling had a higher morbidity and mortality risk than WEB consistent with the data retrieved from the selected studies. However, one might argue that morbidity and mortality rates for the WEB might be underestimated, which is often the case in studies on novel techniques or devices (optimism/reporting bias) [27]. To counteract this bias, an additional structural sensitivity analysis was conducted with the same rates applied for morbidity and mortality risks for coiling and WEB.

The key output of PSA is the proportion of results that are cost-effective in relation to a given willingness-to-pay (WTP) threshold (i.e., the maximum amount a decision-maker is willing to pay for one QALY) [5].

## Results

### Base Case Results

In the base case, health benefits were 13.24 QALYs for the WEB, 12.92 for SAC, and 12.68 for coiling. Lifetime costs were 23,167 € in the SAC group, 20,440 € in the WEB group and 8200 € in the coiling group. The ICER for the WEB was 21,826 € per QALY compared to coiling, while SAC was absolutely dominated by WEB (i.e., SAC is costlier and results in less health benefits than WEB).

Regarding neurologic outcome, the cost-effectiveness analysis demonstrated that patients lived without neurologic morbidity for 14.95 years following WEB treatment, 14.50 years following coiling and14.38 years following SAC. Hence SAC was surpassed by the other two strategies. Compared to coiling, the ICER for the WEB was 27,112 € per year of neurologic morbidity avoided. Table 2 shows the cost-effectiveness and cost-utility rankings for the three EVT strategies ranked in order of increasing costs, and the respective ICERs calculated compared to the next cheapest strategy.

**Table 2:** Cost-effectiveness results of the base-case scenario

Strategy	Cost (€)	Incremental Cost (€)	Effectiveness		Incremental Effectiveness		ICER	
			QALY	Neurologic morbidity avoided†	QALY	Neurologic morbidity avoided†	€/QALY	€/neurologic morbidity avoided^a^
Coiling	8200	–	12.68	14.50	–	–	–	–
WEB	20,440	12,240	13.24	14.95	0.56	0.45	21,826	27,112
SAC	23,167	2727	12.92	14.38	−0.32	−0.57	Dominated	Dominated

*WEB* Woven Endobridge, *SAC* stent-assisted coiling, *ICER* incremental cost-effectiveness ratio, *QALY* quality-adjusted life years

^a^Years lived without neurologic morbidity (mRS > 2)

### Sensitivity Analyses

**Fig. 2:**
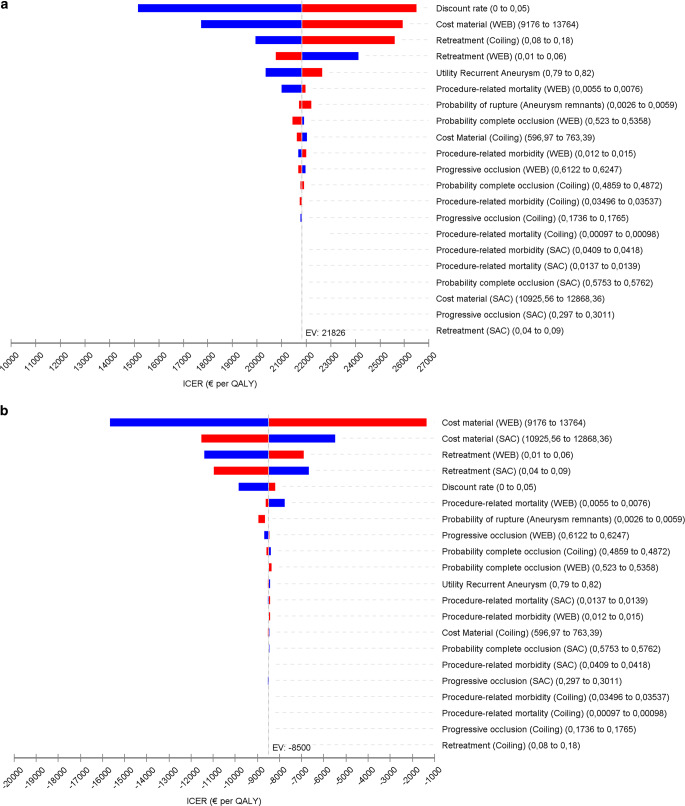
Tornado diagram showing results of deterministic sensitivity analysis for the comparisons (**a**) Coiling vs Woven Endobridge (WEB) and (**b**) stent-assisted coiling (SAC) vs WEB (*ICER* incremental cost effectiveness ratio, *QALY* quality-adjusted life years)

In the deterministic sensitivity analysis, the variables with the largest impact on the ICER were the discount rate, material costs and retreatment rates. In particular, varying the material costs for WEB and SAC affected the ICERs considerably. For instance, considering a 20% higher material cost for WEB, the ICER for the comparison WEB vs. coiling increases 20%, and the ICER for the comparison WEB vs. SAC decreases by 80%. In contrast, considering lower material costs for coiling (e.g. for the case of smaller aneurysms), the ICER for WEB vs. coiling was almost unchanged. Furthermore, considering lower utility values for recurrent/remnant aneurysms (i.e. being at risk for aneurysm rupture has a greater impact on the patient’s perception of quality of life) decreases the ICER for WEB vs. coiling by 7% (20,360 €/QALY). Lastly, varying the probabilities of complete/progressive occlusion of the WEB impacted the ICERs in less than 10%. Figure 2a, b show the results of the deterministic sensitivity analyses for all variables. Note that in all deterministic sensitivity analyses the base case results were confirmed, meaning that WEB has a slightly more favorable cost-effectiveness profile compared to SAC (Supplementary Table S5).

Regarding intracranial stent costs, a reduction of 83% in SAC material costs (1982 € vs. 11,897 €) would be needed to compensate the lower QALY gain for achieving the same cost-effectiveness compared to WEB (Supplementary Table S6 and Fig. S3).

In the structural sensitivity analysis, considering the same morbidity and mortality risk for WEB and coiling, WEB generated 0.45 additional QALYs and costed 13,108 € more than coiling. The resulting ICER for the comparison WEB vs. Coiling was 29,089 €/QALY (30% higher than the base case).

**Fig. 3:**
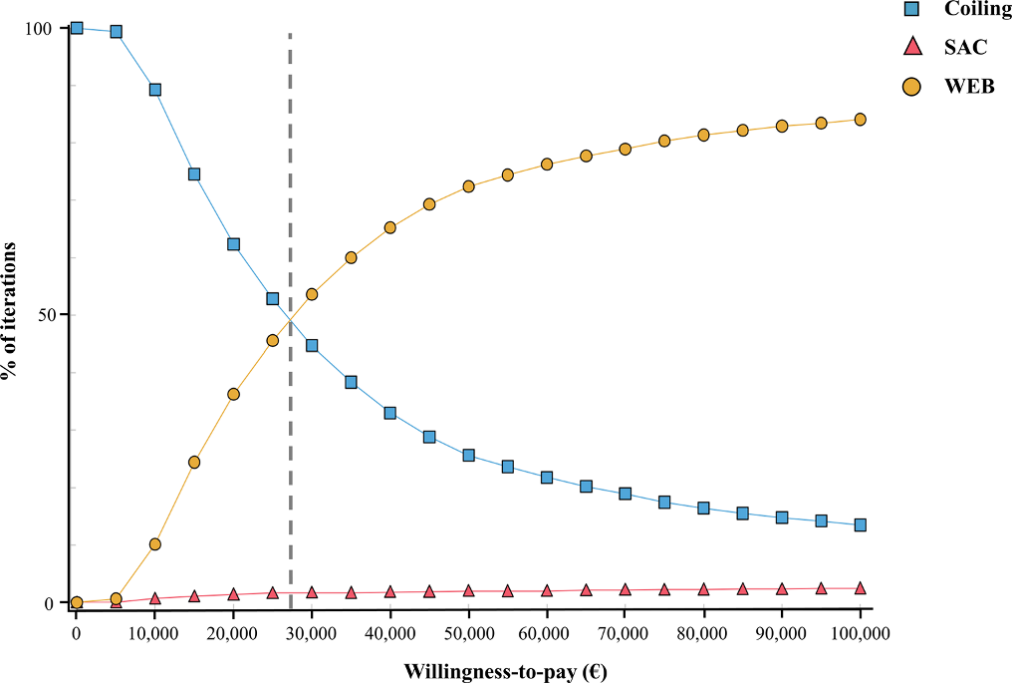
Cost-effectiveness acceptability curve. Willingness-to-pay in € per QALY (quality-adjusted life years) (*WEB* Woven Endobridge, *SAC* stent-assisted coiling)

Supplemental Fig. S2 shows show the results for the PSA plotted in the cost-effectiveness plane. At a WTP of 20,000 €/QALY, the more cost-effective strategy is coiling (with 62% probability); while at a WTP of 30,000 €/QALY, the WEB strategy is superior (with 53% probability). At WTP thresholds higher than 30,000 €/QALY, WEB remains the most cost-effective strategy with increasing certainty (Fig. 3). The probability that SAC is the most cost-effective strategy is less than 2% for all WTP thresholds.

## Discussion

In our model calculations, overall treatment costs (including follow-up examinations and potential rehabilitation costs) of the WEB (20,440 €) were slightly lower than that of SAC (23,167 €), while conventional coiling (8200 €) was the least expensive treatment option. Regarding health outcomes, WEB (13.24 years) and SAC (12.92 years) provided slightly more QALYs than coiling (12.68 years).

Notably, net material costs of WEB and SAC were comparable (11,470 € vs. 11,897 €) for the analysed subset of aneurysms (3–11 mm). Likewise, Kashkoush reported similar treatment costs for WEB ($ 18,530) and SAC ($ 18,950) [23], while Rai et al. reported lower implant costs for the WEB ($ 17,028) than for SAC ($ 23,813) [32]. The slightly increased lifetime costs of SAC over WEB may be largely ascribed to higher rehabilitation costs in the SAC group resulting from a slightly higher initial morbidity (mRS ≥ 2) for SAC compared to WEB (4.1% versus 1.3%). In contrast, the SAC material cost is not a driver of the lack of cost-effectiveness of the SAC strategy. In our analysis, a reduction in SAC material costs of 83% would be required to attain the same cost-effectiveness than WEB treatment. The initial net material costs of coiling (680 €) are distinctly lower than that of WEB or SAC, however, the comparatively high recanalization and retreatment rates increase the overall costs of this procedure. Nevertheless, overall treatment costs of coiling were the lowest.

Although SAC is associated with a higher complication rate and a higher procedure-related morbidity rate than coiling [30], the gains of QALYs were similar for both modalities (12.92 vs. 12.68 years). We attribute this effect by an increased recurrence rate and hence a higher risk of rupture of recanalized aneurysms after coiling compared to SAC.

Results from PSA showed that WEB is potentially cost-effective at WTP thresholds of 30,000 €/QALY gained or higher, while at lower WTP coiling would be the preferred alternative. The results were presented for a range of WTP values, because in Germany there is no commonly accepted threshold [15]. Previous cost-effectiveness analyses assessing interventions aimed at patients with UIA in other settings have applied country-specific WTP thresholds, namely $ 100,000/QALY for the United States, 20,000 to 30,000 £/QALY for the United Kingdom, and 80,000 €/QALY for the Netherlands [6].

Although the hospital perspective was not the focus of our analyses, it should be noted that the same DRG codes are used for WEB and SAC, and therefore, the reimbursed lump-sums are in principle the same [37]. However, material costs for WEB and SAC are covered separately though innovation payments. This financing mechanism was implemented in Germany to promote faster adoption of potentially beneficial innovative medical devices. It is used for DRG-tariffs which are currently not cost-covering for the hospital [10], thereby preventing the provision of innovative devices (e.g., WEB and SAC).

When interpreting the results, it has to be kept in mind that the findings of the current study are only valid for saccular aneurysms between 3 and 11 mm in diameter. Indeed, the indication for the three evaluated modalities differ. In clinical practice, stand-alone coiling is predominantly used for small and large aneurysms with a favorable dome-to-neck ratio. The WEB is suitable for both wide- and narrow-necked aneurysms, however, it is restricted to a saccular shape and an aneurysm diameter ≤ 11 mm [16]. SAC is a well-established treatment option for complex aneurysms including very large, fusiform, lobulated and partially thrombosed aneurysms and vessel branches arising from the aneurysm sac [40]. All these aneurysm types are difficult to treat with the other two modalities and justify SAC as primary treatment for these aneurysms.

The costs of coiling and stent-assisted coiling vary depending on the size of the aneurysm. Smaller sized aneurysms may have lower net material costs, mainly due to a reduced number of coils, while larger aneurysms may be also treated by two overlapping stents. These specifics were considered in our analysis, as we retrospectively recorded the number of implanted devices in aneurysms ranging between 3 and 11 mm. For the WEB, the net material costs would remain stable assuming the implantation of a single device with the appropriate dimensions.

Although newer multi-center studies were available for the WEB, such as the WorldWideWEB Consortium [7], we selected the prospective benchmark studies by Pierot et al. since most WEB studies report on ruptured and unruptured aneurysms and do not differentiate outcome parameters [44]. In the studies by Pierot et al. the portion of ruptured aneurysms was only 8 and 51% were MCA aneurysms. Among high-quality WEB studies, these features fitted to our model best. Nevertheless, the angiographic results of the WorldWideWEB Consortium were also within the range that was considered in our deterministic sensitivity analysis, which led basically to the same results.

Although the WEB is associated with a reasonable safety profile in numerous studies and in our own experience, the complication and morbidity rates may be underestimated in studies of novel interventions or therapies (optimism/reporting bias) [27]. In this context, the reported procedural morbidity of WEB treatment in several studies is lower than that of coiling in the employed meta-analysis by Phan et al. [30] To account for this potential bias, we performed a structural sensitivity analysis assuming similar morbidity rates for coiling and WEB, which showed comparable results to our base case scenario.

### Limitations

Although we performed this study with utmost methodological care, model-based cost-effectiveness studies have several inherent limitations. As the base case scenario was established from the perspective of the German SHI, generalization of the results can be difficult, in particular for different countries and health care systems with diverging reimbursement policy. In this context, material and overall treatment costs can also differ markedly between hospitals in the same country, mainly depending on the purchasing pools and discount contracts between individual hospitals, insurances and manufacturers.

The primary outcomes of this study are based on the results of prior studies that included aneurysms with different location, size and morphology and diverging patient characteristics. Therefore, the applied data may not completely apply to our base case scenario as there might be differences in QALYs, life expectancies, treatment risks and angiographic outcomes. The outcome data for SAC and coiling were mainly derived from a large meta-analysis of predominantly retrospective single-center studies, while the data for the WEB were recorded from three prospective multi-center studies. Admittedly, there are several reviews and meta-analyses on the WEB device, however, these did not report clinical and angiographic outcome for ruptured and unruptured aneurysms separately, which would distort outcome results for elective WEB treatment. In this context, the long-term efficacy of the treatment modalities are difficult to determine, in particular for WEB, for which long-term studies are still rare. At least, the employed study on the WEB report 2‑ and 3‑year angiographic results, which were included into this analysis.

The recruitment periods of the employed WEB studies were between 2011 and 2015. Hence, they did not fully cover recent advances in WEB technology and improvement of WEB handling (e.g. learning curve, +1/−1 rule), which might contribute to slightly better cost-effectiveness results. In this context, the employed meta-analysis on SAC included largely studies on conventional intracranial stent. More advanced stents can have a more flexible structure and a surface finishing designed to reduce device thrombogenicity, which might also increase the attractiveness of SAC from a cost-effectiveness perspective.

Another important limitation was that we adopted coiling for retreatment for all modalities due to the lack of relevant systematic studies. However, in clinical practice, retreatment after WEB implantation is more often performed with an additional stent, whereas retreatment after coiling and SAC is mainly performed by simple recoiling [22, 38]. Retreatment with stents is naturally associated with increased retreatment costs and thromboembolic complications. However, as we assumed a rather low retreatment rate for WEB (3.4%) and SAC (6.2%) in our base case scenario, the main conclusions of this study are probably not significantly distorted by this simplified assumption.

Finally, diverging management strategies (i.e., aneurysm treatment and follow up regimen) and different health care systems might result in different cost-effectiveness of the individual endovascular treatment options. Although the generalizability of the base case result might be limited, we report enough methodological detail to allow adapting this model to other settings.

Despite these limitations, the findings of this study indicate that WEB treatment is a cost-effective treatment alternative to SAC for wide necked aneurysms. The results from our study can be considered in the setting of competing endovascular options for a patient with a standard saccular-shaped unruptured aneurysm to guide an informed decision that optimizes quality of life at affordable costs of intervention and rehabilitation.

## Conclusions

With the increasing use of the WEB for endovascular aneurysm treatment, considering its underlying cost-effectiveness becomes relevant for both health care decision makers and health care providers. In our model calculations, WEB treatment was slightly more cost-effective than SAC. A willingness-to-pay of 30,000 € per QALY gained or higher would be required to justify WEB treatment over coiling. When transferring the results of the present studies to other settings, potential divergences to our base case settings have to be taken into account, such as treatment and follow-up regimen, reimbursement policy and discount contracts.

All studies mentioned were in accordance with the ethical standards indicated in each case.

### Supplementary Information

Supplementary data includes additional information on the input data, as well as the results of the deterministic and probabilistic sensitivity analyses.
