# Supplementary material for: Cost-effectiveness of WEB Embolization, Coiling and Stent-assisted Coiling for the Treatment of Unruptured Intracranial Aneurysms
Source: Clin Neuroradiol. 2023 Jun 27;33(4):1075–86. doi: 10.1007/s00062-023-01311-0 (PMC10654202; doi:10.1007/s00062-023-01311-0)
Supplement: Supplementary file 1 — Supplementary data includes additional information on the input data, as well as the results of the deterministic and probabilistic sensitivity analyses. [file 62_2023_1311_MOESM1_ESM.docx]

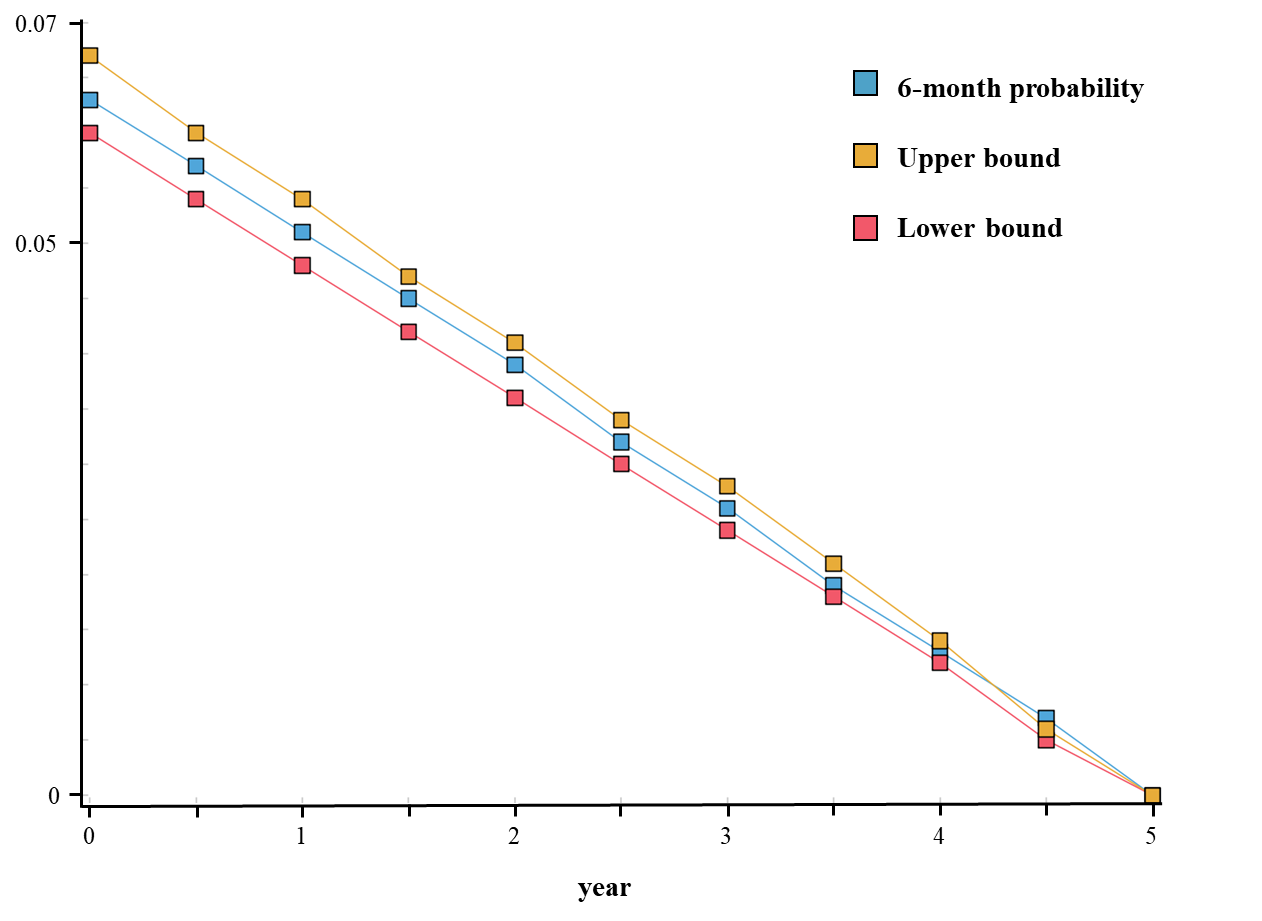


**Fig. S1** Assumption of decreasing recurrence rate for the first 5 years for WEB. The same assumption was made for SAC and Coiling.

**Table S1**. Lifelong probability of retreatment per strategy. SAC = stent-assisted coiling, WEB = Woven Endobridge.

| Strategies | Lifelong cumulative probability of retreatment | Lifelong cumulative probability of second retreatment |
| --- | --- | --- |
| Coiling | 0,2828 | 0,0632 |
| SAC | 0,2084 | 0,0508 |
| WEB | 0,1843 | 0,0633 |

**Table S2.** Resource consumption of materials and total material costs. SAC = stent-assisted coiling, WEB = Woven Endobridge. SD = standard deviation.

|  | Patients (n) | Aneurysms (n) | Coils per aneurysm  Mean (SD) | Stents per aneurysm  Mean (SD) | Unitary cost per coil † | Unitary cost per Stent † | Unitary cost per WEB device† | Total material costs  Mean (SD) |
| --- | --- | --- | --- | --- | --- | --- | --- | --- |
| Coiling | 80 | 81 | 4,8 (2,6) | - | € 142 |  |  | € 680 (376) |
| SAC | 57 | 63 | 5,08 (2,97) | 1,14 (0,35) | € 142 | €9,803 |  | € 11,896 (3857) |
| WEB ^§^ | - | - | - | - |  |  | € 11,470 | € 11,470 (2294) |

^§^ WEB costs: Assumption of one WEB device per aneurysm; and a standard deviation of 20%. †Unitary costs were obtained from the author’s institution.

**Table S3.** Diagnosis-related group (DRG) reimbursement lump-sums. SAC = stent-assisted coiling, WEB = Woven Endobridgem, OPS = operations and procedures key (German: “*Operationen- und Prozedurenschlüssel*”)

|  | DRG / OPS code | Duration of hospital stay (days) | Reimbursement lump-sum |
| --- | --- | --- | --- |
| Coiling | B81B/ 8-83b.30 | 1 | 1059.32 |
|  |  | 2 | 2513.12 |
|  |  | 3 | 2513.12 |
| WEB or SAC | B20D / 8-83c.8 | 1 | 4959.69 |
|  |  | 2 | 6633.24 |
|  |  | 3 | 8306.79 |

**Table S4** Parameters used for the probabilistic sensitivity analysis. SAC = stent-assisted coiling, WEB = Woven Endobridge, mRS = modified Rankin scale, SAH = subarachnoid hemorrhage.

| Parameter | Parameters | Distribution | Source |
| --- | --- | --- | --- |
| Event probabilities | (α, β) |  |  |
| WEB |  |  |  |
| Procedure morbidity (leading up to mRS score>2) | (1.97; 149.28) | β | [[1](#_ENREF_1)] |
| Procedure-related mortality | (1.14; 161.87) | β | [[1](#_ENREF_1)] |
| Adequate occlusion after procedure | (80.4; 71.6) | β | [[1](#_ENREF_1)] |
| Progressive occlusion | (93.38; 57.62) | β | [[1](#_ENREF_1)] |
| Retreatment | (1.39; 39.68) | β | [[1](#_ENREF_1)] |
| SAC |  | β |  |
| Procedure morbidity (leading up to mRS score>2) | (32.39; 757.69) | β | [[2](#_ENREF_2)] |
| Procedure-related mortality | (30.03; 2146.49) | β | [[2](#_ENREF_2)] |
| Adequate occlusion after procedure | (1228.11; 904.03) | β | [[2](#_ENREF_2)] |
| Progressive occlusion | (130.60; 306.21) | β | [[2](#_ENREF_2)] |
| Retreatment | (52.32; 771.69) | β | [[3](#_ENREF_3),[4](#_ENREF_4)] |
| Coiling |  | β |  |
| Procedure morbidity (leading up to mRS score>2) | (61.30; 1690.22) | β | [[2](#_ENREF_2)] |
| Procedure-related mortality | (61.59; 27936.89)) | β | [[2](#_ENREF_2)] |
| Adequate occlusion after procedure | (778.78; 819.73) | β | [[2](#_ENREF_2)] |
| Progressive occlusion in 6 months | (89.86; 423.34) | β | [[2](#_ENREF_2)] |
| Retreatment | (156.58; 1046.03) | β | [[4](#_ENREF_4)] |
| Retreatment (coiling for all groups) |  | β |  |
| Procedure morbidity (leading up to mRS score >2) | 0.027 (0.015) | β | [[5](#_ENREF_5)] |
| Procedure-related mortality | 0.001 (0.0001) | β | [[2](#_ENREF_2)] |
| Adequate occlusion after procedure | 0.487 (0.012) | β | [[2](#_ENREF_2)] |
| Probability of a second retreatment | 0.025 (0.015) | β | [[5](#_ENREF_5)] |
| Probability of rupture of remnants or recurrent aneurysm | (0.03; 37.37) | β | [[6](#_ENREF_6)] |
| Probability of poor outcome after rupture (mRS 3-5) | (290.09; 1315.31) | β | [[6](#_ENREF_6)] |
| SAH mortality | (149.23; 1490.69) | β | [[6](#_ENREF_6)] |
| Probability of bleeding due to long-term low dose aspirin | (1.45; 399.41) |  | [[7](#_ENREF_7)] |
| Costs | (α, λ) |  |  |
| WEB material costs | (25; 0.002) | γ |  |
| SAC material costs | (9.51; 7.99) | γ |  |
| Coiling material costs | (3.26; 0.0048) | γ |  |
| Acute in-hospital SAH treatment | (25; 0.001) | γ | [[8](#_ENREF_8)] |
| Consequential in-hospital SAH treatment | (25; 0.006) | γ | [[8](#_ENREF_8)] |
| Rehabilitation | (25; 0.002) | γ | [[8](#_ENREF_8)] |
| Home care costs | (25; 0.002) | γ | [[8](#_ENREF_8)] |
| Digital subtraction angiography (inpatient procedure) ‡ | (24.81; 0.09) | γ | [[9](#_ENREF_9)] |
| Magnetic resonance angiography (outpatient procedure) ‡ | (25; 0.32) | γ | [[9](#_ENREF_9)] |
| Utilities | (α, β) |  |  |
| Age-adjusted utility values for healthy population (55 years) | (6.10; 0.32) | β | [[10](#_ENREF_10)] |
| Utility values for harboring an untreated aneurysm | (2.64;0.61) | β | [[11](#_ENREF_11)] |
| Utility decrements for bleeding events associated with antiplatelet therapy | (0.34; 11.16) | β | [[12](#_ENREF_12)] |
| Utility values after SAH according to outcome  Good outcome  mRS 0  mRS 1  mRS 2  Bad outcome  mRS 3  mRS 4  mRS 5 | (1.76;0.13)  (3.74; 0.60)  (3.01;1.42)  (2.04;1.60)  (0.75; 1.67)  (0.01;0.26) | β | [[13](#_ENREF_13)] |


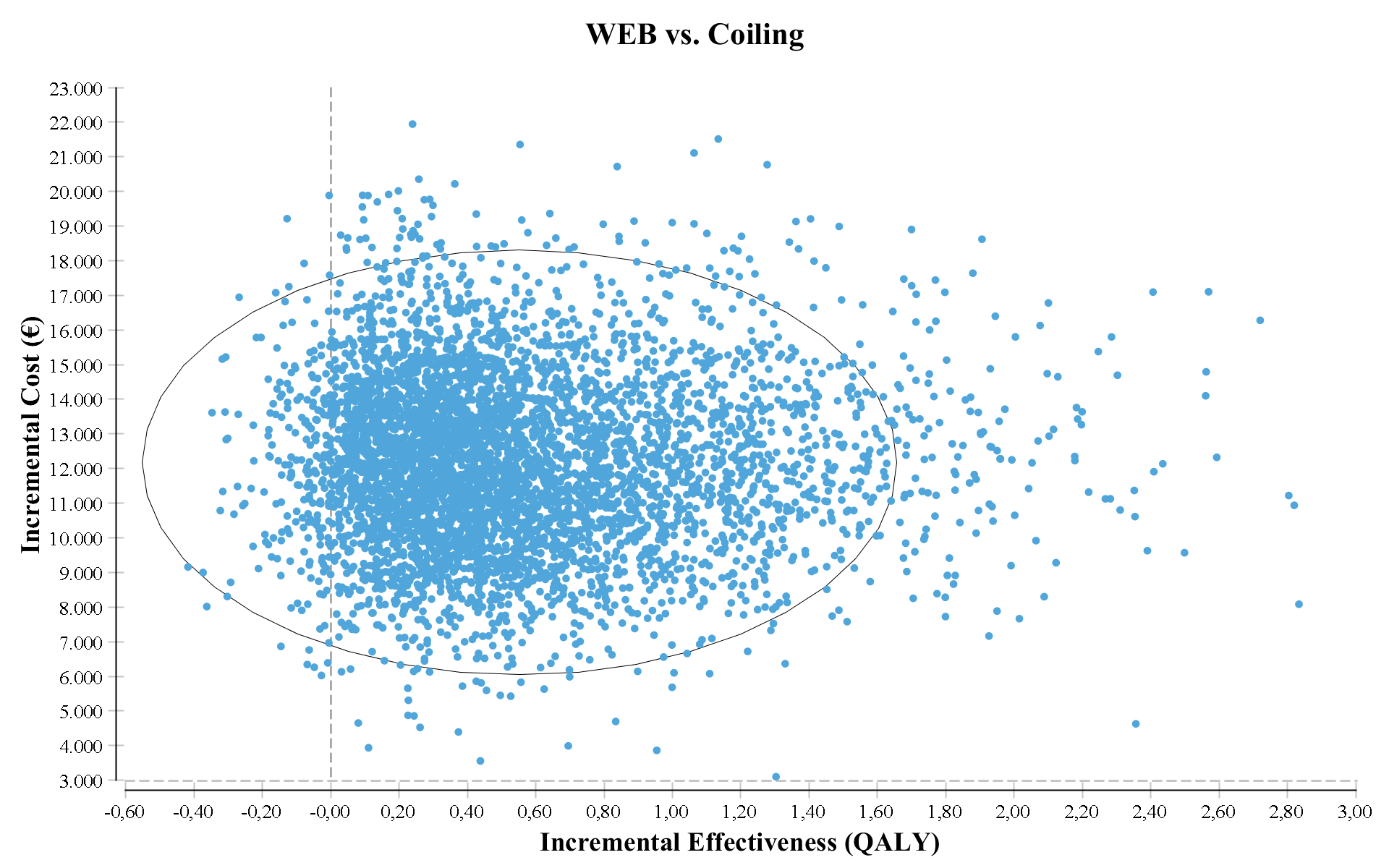


(A)


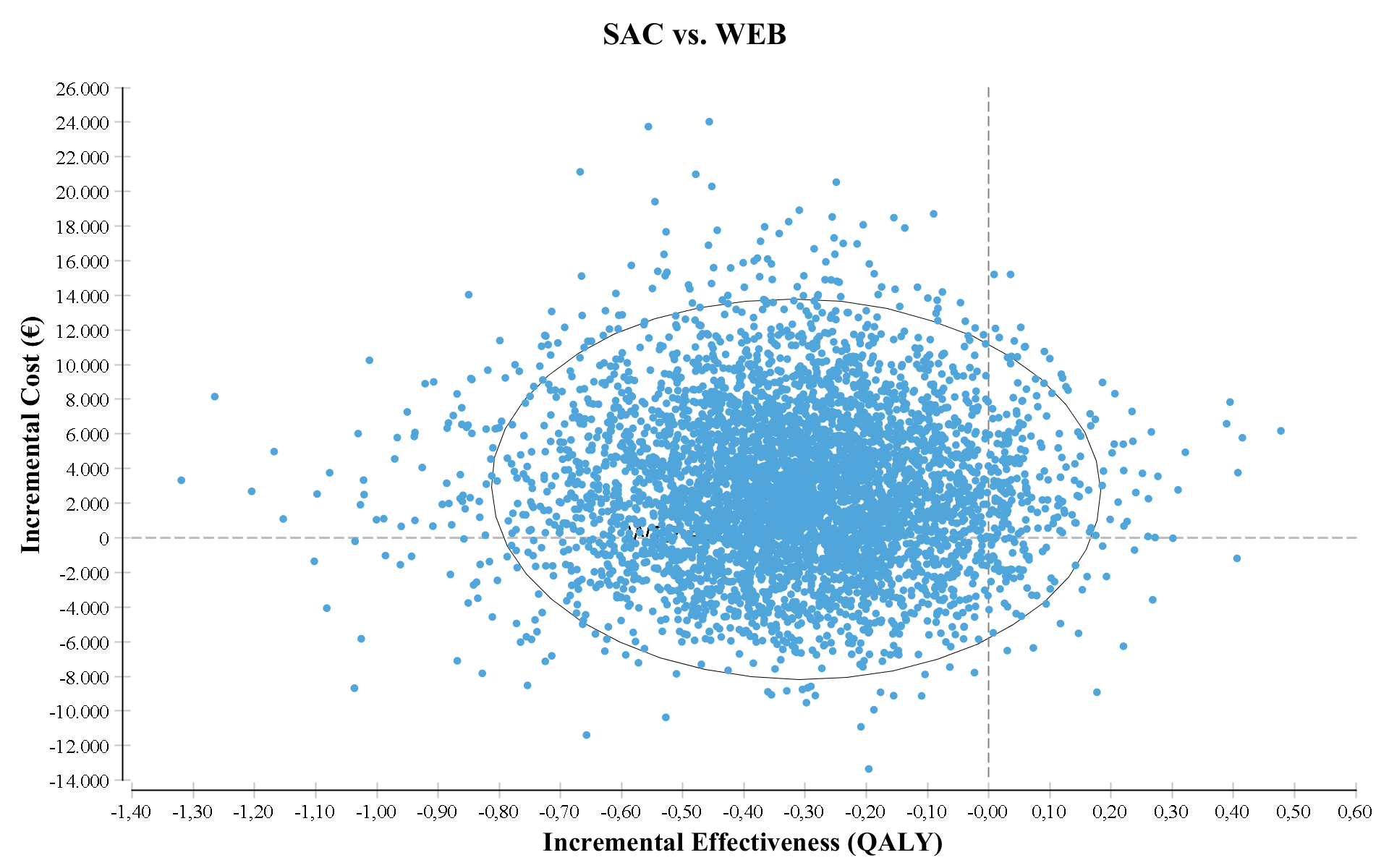
(B)

**Fig. S2** Results of probabilistic sensitivity analysis plotted in the cost-effectiveness plane for the comparisons (A) Woven Endobridge (WEB) vs. Coiling and (B) stent-assisted coiling (SAC) vs. WEB.

**Table S5** Cost-effectiveness rankings resulting from deterministic sensitivity analyses. SAC = stent-assisted coiling, WEB = Woven Endobridge, ICER: incremental cost effectiveness ratio, QALY: quality-adjusted life years. Incremental values calculated in relation to the strategy immediately above.

| Input parameter | Strategy | Cost (€) | Incremental Cost (€) | Effectiveness (QALY) | Incremental effectiveness (QALY) | ICER €/QALY |  |
| --- | --- | --- | --- | --- | --- | --- | --- |
| Discount rate |  |  |  |  |  |  |  |
| Lower value: 0% | Coiling | 10438 | - | 18.98 | - | - |  |
|  | WEB | 22332 | 11859 | 19.76 | 0.78 | 15146 |  |
|  | SAC | 26337 | 4004 | 19.35 | -0.41 | -9848 | Dominated |
| Upper value: 5% | Coiling | 7363 | - | 10.2 | - | - |  |
|  | WEB | 19720 | 12357 | 10.67 | 0.47 | 26497 |  |
|  | SAC | 21983 | 2263 | 10.39 | -0.28 | -8182 | Dominated |
| Probability of remnant rupture | |  |  |  |  |  |  |
| Lower value: 0.0041 | Coiling | 7552 | - | 12.7 | - | - |  |
|  | WEB | 19974 | 12422 | 13.25 | 0.56 | 22332 |  |
|  | SAC | 22680 | 2705 | 12.94 | -0.31 | -8677 | Dominated |
| Upper value: 0.0138 | Coiling | 9249 | - | 12.71 | - | - |  |
|  | WEB | 21184 | 11934 | 13.25 | 0.54 | 22122 |  |
|  | SAC | 23898 | 2717 | 12.94 | -0.32 | -8645 | Dominated |
| Utility recurrent aneurysm | |  |  |  |  |  |  |
| Lower value: 0.79 | Coiling | 8200 | - | 12.53 | - | - |  |
|  | WEB | 20440 | 12240 | 13.13 | 0.60 | 20360 |  |
|  | SAC | 23167 | 2726 | 12.81 | -0.32 | -8442 | Dominated |
| Upper value: 0.82 | Coiling | 8200 | - | 12.75 | - | - |  |
|  | WEB | 20440 | 12240 | 13.29 | 0.54 | 22642 |  |
|  | SAC | 23167 | 2726 | 12.97 | -0.32 | -8530 | Dominated |
| Cost of WEB material | |  |  |  |  |  |  |
| Lower value:  9176 € | Coiling | 8200 | - | 12.68 | - | - |  |
|  | WEB | 18146 | 9946 | 13.24 | 0.56 | 17736 |  |
|  | SAC | 23167 | 5021 | 12.92 | -0.32 | -15652 | Dominated |
| Upper value:  13764 € | Coiling | 8200 | - | 12.68 | - | - |  |
|  | WEB | 22734 | 14534 | 13.24 | 0.56 | 25971 |  |
|  | SAC | 23167 | 433 | 12.92 | -0.32 | -1350 | Dominated |
| Cost of SAC material | |  |  |  |  |  |  |
| Lower value:  10925 € | Coiling | 8200 | - | 12.68 | - | - |  |
|  | WEB | 20440 | 12240 | 13.24 | 0.56 | 21826 |  |
|  | SAC | 22196 | 1756 | 12.92 | -0.32 | -5476 | Dominated |
| Upper value:  12868 € | Coiling | 8200 | - | 12.68 | - | - |  |
|  | WEB | 20440 | 12240 | 13.24 | 0.56 | 21826 |  |
|  | SAC | 12868 | 3698 | 12.92 | -0.32 | -11529 | Dominated |
| Cost of Coiling Material | |  |  |  |  |  |  |
| Lower value: 597 € | Coiling | 8078 | - | 12.68 | - | - |  |
|  | WEB | 20433 | 12355 | 13.24 | 0.56 | 22031 |  |
|  | SAC | 23152 | 2719 | 12.92 | -0.32 | -8476 | Dominated |
| Upper value: 763 € | Coiling | 8323 | - | 12.68 | - | - |  |
|  | WEB | 20448 | 12125 | 13.24 | 0.56 | 21622 |  |
|  | SAC | 23183 | 2735 | 12.92 | -0.32 | -8525 | Dominated |
| Retreatment Rate Coiling | |  |  |  |  |  |  |
| Lower value: 0.08 | Coiling | 7665 | - | 12.60 | - | - |  |
|  | WEB | 20440 | 12775 | 13.24 | 0.64 | 19926 |  |
|  | SAC | 23167 | 2727 | 12.92 | -0.32 | -8501 | Dominated |
| Upper value: 0.18 | Coiling | 8561 |  | 12.78 | - | - |  |
|  | WEB | 20440 | 11879 | 13.24 | 0.46 | 25599 |  |
|  | SAC | 23167 | 2727 | 12.92 | -0.32 | -8501 | Dominated |
| Retreatment Rate SAC | |  |  |  |  |  |  |
| Lower value: 0,05 | Coiling | 8200 | - | 12.68 | - | - |  |
|  | WEB | 20440 | 12240 | 13.24 | 0.56 | 21826 |  |
|  | SAC | 22954 | 2513 | 12.86 | -0.38 | -6664 | Dominated |
| Upper value: 0,09 | Coiling | 8200 | - | 12.68 | - | - |  |
|  | WEB | 20440 | 12240 | 13.24 | 0.56 | 21826 |  |
|  | SAC | 23364 | 2924 | 12.97 | -0.27 | -10957 | Dominated |
| Retreatment Rate WEB | |  |  |  |  |  |  |
| Lower value: 0,01 | Coiling | 8200 | - | 12.68 | - | - |  |
|  | WEB | 20168 | 11968 | 13.15 | 0.47 | 25464 |  |
|  | SAC | 23167 | 2999 | 12,92 | -0.23 | -13039 | Dominated |
| Upper value: 0,06 | Coiling | 8200 | - | 12.68 | - | - |  |
|  | WEB | 20636 | 12436 | 13.27 | 0.59 | 21078 |  |
|  | SAC | 23167 | 2531 | 12.92 | -0.35 | -7232 | Dominated |

**Table S6** Cost-effectiveness rankings resulting from structural sensitivity analyses. SAC = stent-assisted coiling, WEB = Woven Endobridge, ICER: incremental cost effectiveness ratio, QALY: quality-adjusted life years.

| Input parameter | Strategy | Cost (€) | Incremental Cost (€) | Effectiveness (QALY) | Incremental effectiveness (QALY) | ICER €/QALY |  |
| --- | --- | --- | --- | --- | --- | --- | --- |
| ^a^ Would higher mortality and morbidity risks of WEB impact the results? | | | | | | | |
| Morbidity and mortality risks of coiling applied to WEB | Coiling | 8188 | - | 12.67 | - | - |  |
|  | WEB | 21296 | 13108 | 13.12 | 0.45 | 29089 |  |
|  | SAC | 23164 | 1868 | 12.91 | -0.21 | -8816 | Dominated |
| How would identical retreatment risks impact the results? | | | | | | | |
| Assuming identical retreatment risks in all groups | Coiling | 8207 | - | 12.66 | - | - |  |
|  | WEB | 21040 | 12833 | 13.35 | 0.70 | 18438 |  |
|  | SAC | 23570 | 15363 | 13.01 | 0.35 | 43365 | Dominated |
| ^b^ Would lower SAC material costs impact the results? | | | | | | | |
| Assuming 50% reduction on SAC material costs | Coiling | 8,200 | - | 12.68 | - | - |  |
|  | SAC | 17,219 | 9,018 | 12.92 | 0.24 | 37576 | Ext. Dominated |
|  | WEB | 20,440 | 12,240 | 13.24 | 0.56 | 21826 |  |
| Assuming 83% reduction on SAC material costs | Coiling | 8,200 | - | 12.68 | - | - |  |
|  | SAC | 13,253 | 5,052 | 12.92 | 0.24 | 21,053 | Ext. Dominated |
|  | WEB | 20,440 | 12,240 | 13.24 | 0.56 | 21826 |  |
| If sizing is not correct and a second WEB device is used in 20% of cases, how would this impact the results? | | | | | | | |
| Assuming 20% of patients would need a second WEB | Coiling | 8200 | - | 12.68 | - | - |  |
|  | WEB | 22734 | 14,534 | 13.24 | 0.56 | 25,917 |  |
|  | SAC | 23167 | 14,967 | 12.92 | 0.24 | 62,361 | Dominated |

^a^ Structural sensitivity analysis determined a priori. Other structural sensitivity analyses were tested in view of results during the peer review process.

^b^ These results were plotted in the cost-effectiveness plane for better visualization (Go to Figure S3). When we consider 50% reduction in SAC material costs, SAC is still dominated by the two other strategies, because WEB still generates higher QALYs. To compensate the lower QALY gain (in contrast with the QALY gain provided by WEB), a reduction of 83% in SAC material costs would be needed (i.e., SAC costs would have to cost € 1982 instead of €11,897 to beat WEB in terms of cost-effectiveness)


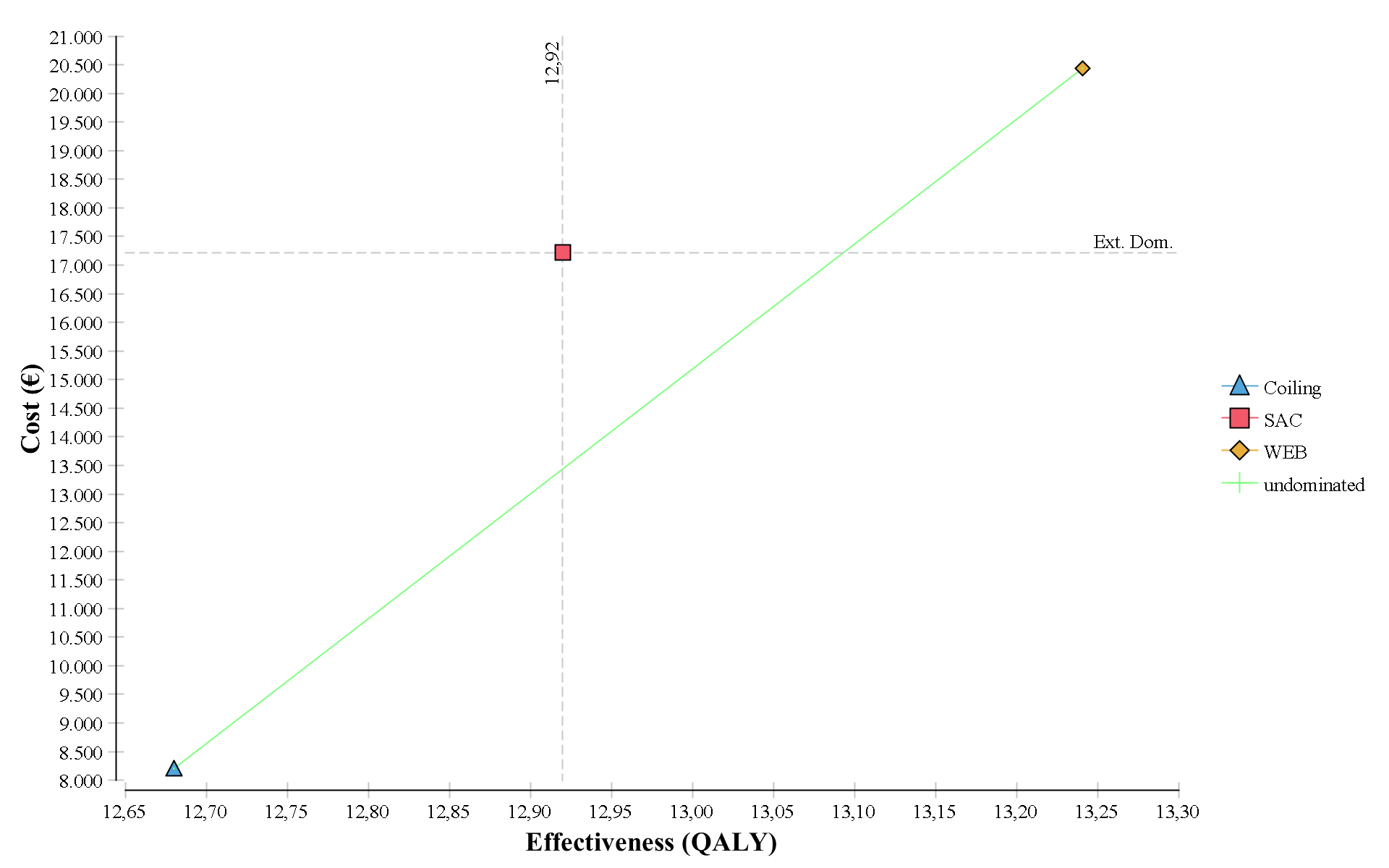


**Figure S3.** Costs in function of effectiveness assuming a 50% reduction on SAC material costs. SAC = stent-assisted coiling, WEB = Woven Endobridge, QALY: quality-adjusted life years.

*Assessment of the Validation Status of Health Economic decision models (AdViSHE)*

The validation process of the model is reported below according to the questions of the AdViSHE checklist [[14](#_ENREF_14)], which is divided in 4 parts:

**Part A: Validation of the conceptual model (2 questions)**

| A1/ Face validity testing (conceptual model): Have experts been asked to judge the appropriateness of the conceptual model? |
| --- |
| Experts with different backgrounds and expertise were asked to judge the appropriateness of the conceptual model.  One issue raised was that it is difficult to compare the three treatment options, because they are not easily interchangeable especially primary coiling versus SAC or WEB. Hence, we agreed on a target population that adequately reflects the patient to whom WEB is mostly indicated (single UIA located at the middle cerebral artery (MCA) with saccular shape and an aneurysm diameter between 3 and 11 mm). |

| A2/ Cross validity testing (conceptual model): Has this model been compared to other conceptual models found in the literature or clinical textbooks? |
| --- |
| The concept of the model was developed based in similar models comparing different strategies to the treatment of bone metastases. For instance, the health states considered are similar and the transitions and equivalent to other Markov models. |

**Part B: Input data validation (2 questions)**

| B1/ Face validity testing (input data): Have experts been asked to judge the appropriateness of the input data? |
| --- |
| Appropriateness of input data was judged by all authors before and after data imputation. |

| B2/ Model fit testing: When input parameters are based on regression models, have statistical tests been performed? |
| --- |
| We adjusted yearly values to fit the model’s 6-month cycles and transformed rates into probabilities. These calculations were done by JSCG and reviewed by a second model expert (DM). |

**Part C: Validation of the computerized model (4 questions)**

| C1/ External review: Has the computerized model been examined by modelling experts? |
| --- |
| Yes. The computerized model was checked by a modelling expert (DM), after data imputation by JSCG. |

| C2/ Extreme value testing: Has the model been run for specific, extreme sets of parameter values in order to detect any coding errors? |
| --- |
| To detect coding errors, we tested extreme values for cost, utilities and event probabilities. All variables were tested in deterministic sensitivity analyses. |

| C3/ Testing of traces: Have patients been tracked through the model to determine whether its logic is correct? |
| --- |
| Yes, we reviewed individual trials to check for if the events occurring during patients’ lifetime were plausible. We used trackers (retreatment and rupture) to identify at cohort level if the model’s logic was correct. |

| C4/ Unit testing: Have individual sub-modules of the computerized model been tested? |
| --- |
| Yes. We tested and reported alternative scenarios in the main manuscript and the supplementary material. |

**Part D: Operational validation (4 questions)**

| D1/ Face validity testing (model outcomes): Have experts been asked to judge the appropriateness of the model outcomes? |
| --- |
| Yes. The appropriateness of model outcomes was judged by all authors. |

| D2/ Cross validation testing (model outcomes): Have the model outcomes been compared to the outcomes of other models that address similar problems? |
| --- |
| Partially done, the model concept and data applied was similar to other models, but there was limited comparability with other models regarding results, since this is the first model to present the comparison WEB vs SAC vs coiling. |

| D3/ Validation against outcomes using alternative input data: Have the model outcomes been compared to the outcomes obtained when using alternative input data? |
| --- |
| Yes. We conducted several structural sensitivity analyses, reported in the main manuscript and in the supplementary material. |

| D4/ Validation against empirical data: Have the model outcomes been compared to empirical data? |
| --- |
| A unit simulating patients that do not undergo either endovascular treatment was used for validation purposes, to guarantee the microsimulation model was able to replicate the available empirical data for patients without treatment. |

**References**

1. Pierot, L.; Moret, J.; Barreau, X.; Szikora, I.; Herbreteau, D.; Turjman, F.; Holtmannspotter, M.; Januel, A.C.; Costalat, V.; Fiehler, J., et al. Safety and efficacy of aneurysm treatment with WEB in the cumulative population of three prospective, multicenter series. *J Neurointerv Surg* **2018**, *10*, 553-559, doi:10.1136/neurintsurg-2017-013448.

2. Phan, K.; Huo, Y.R.; Jia, F.; Phan, S.; Rao, P.J.; Mobbs, R.J.; Mortimer, A.M. Meta-analysis of stent-assisted coiling versus coiling-only for the treatment of intracranial aneurysms. *J Clin Neurosci* **2016**, *31*, 15-22, doi:10.1016/j.jocn.2016.01.035.

3. Fukuda, H.; Sato, D.; Kato, Y.; Tsuruta, W.; Katsumata, M.; Hosoo, H.; Matsumaru, Y.; Yamamoto, T. Comparing Retreatments and Expenditures in Flow Diversion Versus Coiling for Unruptured Intracranial Aneurysm Treatment: A Retrospective Cohort Study Using a Real-World National Database. *Neurosurgery* **2020**, *87*, 63-70, doi:10.1093/neuros/nyz377.

4. Lecler, A.; Raymond, J.; Rodriguez-Régent, C.; Al Shareef, F.; Trystram, D.; Godon-Hardy, S.; Ben Hassen, W.; Meder, J.F.; Oppenheim, C.; Naggara, O.N. Intracranial Aneurysms: Recurrences More than 10 Years after Endovascular Treatment-A Prospective Cohort Study, Systematic Review, and Meta-Analysis. *Radiology* **2015**, *277*, 173-180, doi:10.1148/radiol.2015142496.

5. Teleb, M.S.; Pandya, D.J.; Castonguay, A.C.; Eckardt, G.; Sweis, R.; Lazzaro, M.A.; Issa, M.A.; Fitzsimmons, B.F.; Lynch, J.R.; Zaidat, O.O. Safety and predictors of aneurysm retreatment for remnant intracranial aneurysm after initial endovascular embolization. *J Neurointerv Surg* **2014**, *6*, 490-494, doi:10.1136/neurintsurg-2013-010836.

6. Funakoshi, Y.; Imamura, H.; Tani, S.; Adachi, H.; Fukumitsu, R.; Sunohara, T.; Omura, Y.; Matsui, Y.; Sasaki, N.; Fukuda, T., et al. Predictors of Cerebral Aneurysm Rupture after Coil Embolization: Single-Center Experience with Recanalized Aneurysms. *AJNR Am J Neuroradiol* **2020**, *41*, 828-835, doi:10.3174/ajnr.A6558.

7. García Rodríguez, L.A.; Martín-Pérez, M.; Hennekens, C.H.; Rothwell, P.M.; Lanas, A. Bleeding Risk with Long-Term Low-Dose Aspirin: A Systematic Review of Observational Studies. *PLoS One* **2016**, *11*, e0160046, doi:10.1371/journal.pone.0160046.

8. Ridwan, S.; Urbach, H.; Greschus, S.; von Hagen, J.; Esche, J.; Boström, A. Health Care Costs of Spontaneous Aneurysmal Subarachnoid Hemorrhage for Rehabilitation, Home Care, and In-Hospital Treatment for the First Year. *World Neurosurg* **2017**, *97*, 495-500, doi:10.1016/j.wneu.2016.09.123.

9. Krankenhaus, I.f.E.i. **2022**.

10. Grochtdreis, T.; Dams, J.; König, H.H.; Konnopka, A. Health-related quality of life measured with the EQ-5D-5L: estimation of normative index values based on a representative German population sample and value set. *The European journal of health economics : HEPAC : health economics in prevention and care* **2019**, *20*, 933-944, doi:10.1007/s10198-019-01054-1.

11. King, J.T., Jr.; Brandt, C.A.; Tsevat, J.; Roberts, M.S. A national internet-based survey of cerebral aneurysm preference-based quality of life. *Neurosurgery* **2009**, *64*, 249-254; discussion 254-245, doi:10.1227/01.Neu.0000333266.18738.Be.

12. Doble, B.; Pufulete, M.; Harris, J.M.; Johnson, T.; Lasserson, D.; Reeves, B.C.; Wordsworth, S. Health-related quality of life impact of minor and major bleeding events during dual antiplatelet therapy: a systematic literature review and patient preference elicitation study. *Health Qual Life Outcomes* **2018**, *16*, 191, doi:10.1186/s12955-018-1019-3.

13. Rebchuk, A.D.; O'Neill, Z.R.; Szefer, E.K.; Hill, M.D.; Field, T.S. Health Utility Weighting of the Modified Rankin Scale: A Systematic Review and Meta-analysis. *JAMA Netw Open* **2020**, *3*, e203767, doi:10.1001/jamanetworkopen.2020.3767.

14. Vemer, P.; Corro Ramos, I.; van Voorn, G.A.; Al, M.J.; Feenstra, T.L. AdViSHE: A Validation-Assessment Tool of Health-Economic Models for Decision Makers and Model Users. *Pharmacoeconomics* **2016**, *34*, 349-361, doi:10.1007/s40273-015-0327-2.
